# Authors’ reply

**Published:** 2010

**Authors:** Savitri Sharma, Sujata Das

**Affiliations:** Ocular Microbiology Service, LV Prasad Eye Institute, Bhubaneswar, Orissa, India; 1 Cornea and Anterior Segment Service, LV Prasad Eye Institute, Bhubaneswar, Orissa, India

Dear Editor,

We appreciate the comments of Wiwanitkit[1] on our article.[2] The fact that Sabouraud dextrose agar is the standard medium for fungal culture and identification is irrefutable and the same has been mentioned in our article. We however reiterate our observation that in the case of mycotic keratitis, the diagnosis is unlikely to be missed if only blood or chocolate agar is used since the fungi causing keratitis can grow well on blood and chocolate agar. We agree that certain fungi like *Histoplasma* sp. may not grow on blood or chocolate agar; however, we would like to mention that this group of fungi is normally not associated with fungal keratitis.
